# Chromosomal Instability and Clonal Heterogeneity in Breast Cancer: From Mechanisms to Clinical Applications

**DOI:** 10.3390/cancers17071222

**Published:** 2025-04-04

**Authors:** María Paula Meléndez-Flórez, Oscar Ortega-Recalde, Nelson Rangel, Milena Rondón-Lagos

**Affiliations:** 1 Departamento de Morfología, Facultad de Medicina e Instituto de Genética, Universidad Nacional de Colombia, Bogotá 110231, Colombia; mmelendezf@unal.edu.co (M.P.M.-F.); oortegar@unal.edu.co (O.O.-R.); 2 Department of Pathology, Instituto Nacional de Cancerología, Bogotá 110231, Colombia; 3 Departamento de Nutrición y Bioquímica, Facultad de Ciencias, Pontificia Universidad Javeriana, Bogotá 110231, Colombia; 4 Escuela de Ciencias Biológicas, Universidad Pedagógica y Tecnológica de Colombia, Tunja 150003, Colombia

**Keywords:** chromosomal instability, aneuploidy, clonal heterogeneity, single-cell sequencing, gene expression, breast cancer

## Abstract

Background: Chromosomal instability (CIN) and clonal heterogeneity (CH) are fundamental hallmarks of breast cancer that drive tumor evolution, disease progression, and therapeutic resistance. Understanding the mechanisms underlying these phenomena is essential for improving cancer diagnosis, prognosis, and treatment strategies. Methods: In this review, we provide a comprehensive overview of the biological processes contributing to CIN and CH, highlighting their molecular determinants and clinical relevance. Results: We discuss the latest advances in detection methods, including single-cell sequencing and other high-resolution techniques, which have enhanced our ability to characterize intratumoral heterogeneity. Additionally, we explore how CIN and CH influence treatment responses, their potential as therapeutic targets, and their role in shaping the tumor immune microenvironment, which has implications for immunotherapy effectiveness. Conclusions: By integrating recent findings, this review underscores the impact of CIN and CH on breast cancer progression and their translational implications for precision medicine.

## Simple Summary

Breast cancer is characterized by chromosomal instability (CIN) and clonal heterogeneity (CH), which contribute to tumor progression and treatment resistance. Understanding these processes is essential for improving patient care. In this review, we explore the biological mechanisms driving CIN and CH, their impact on tumor evolution, and their clinical significance. We also discuss advanced detection methods, such as single-cell sequencing, and potential therapeutic strategies targeting CIN and CH. By summarizing recent findings, we highlight their relevance in breast cancer diagnosis, prognosis, and treatment.

## 1. Introduction

Chromosomal instability (CIN), characterized by an increased rate of chromosome missegregation, is a hallmark of cancer [1,2]. This instability leads to aneuploidy and extensive genomic alterations, disrupting critical cellular functions and promoting tumor aggressiveness, disease progression, and therapeutic resistance [3,4]. CIN also drives clonal heterogeneity (CH), resulting in diverse subpopulations of cancer cells within the same tumor, each possessing unique genetic and phenotypic profiles [5,6]. This diversity enables certain clones to adapt and survive under therapeutic pressures, contributing to treatment resistance and recurrence [7]. Both CIN and CH are crucial factors influencing breast cancer (BC) progression, therapeutic resistance, and patient outcomes [8]. In BC, CIN drives tumor heterogeneity and promotes the formation of aggressive subclones with metastatic potential [9]. Notably, some studies have reported that 89% of invasive BC cases exhibit CIN, with levels varying across different subtypes [10,11]. However, a paradoxical relationship has been observed between chromosomal CIN and survival outcomes in BC patients. For example, in a cohort of 265 TNBC patients, those with extreme CIN exhibited a better prognosis, a reduced risk of recurrence, and longer survival times [12]. Similarly, an analysis of 1173 BC patients found that extreme CIN was associated with a better prognosis in estrogen receptor-negative (ER-)/HER2-negative BC patients, suggesting that extreme CIN could serve as a favorable prognostic marker [13]. These findings emphasize the complex relationship between CIN and the prognosis: while tumors with high proliferative and metastatic capacity display some degree of CIN, extreme CIN may result in genomic instability beyond repair, potentially hindering tumor growth [14].

The molecular mechanisms driving CIN remain largely elusive. It has been estimated that approximately 2300 genes can induce CIN through aberrant expression, yet fewer than 150 of these genes have been identified and thoroughly validated [15]. Most of these genes are associated with well-characterized pathways involved in chromosome dynamics and DNA repair, including processes such as chromosome condensation, chromosome segregation, sister chromatid cohesion, telomere maintenance, kinetochore–microtubule attachments, mitotic spindle dynamics, spindle assembly and checkpoint mechanisms, centrosome regulation, and DNA replication and repair [16,17]. Expanding our knowledge of molecular pathways involved in the induction and maintenance of CIN is essential for identifying novel therapeutic targets and refining strategies to predict, prevent, and treat cancer progression. Additionally, growing evidence suggests that CIN and CH influence the tumor immune microenvironment by modulating antigen presentation, immune evasion, and inflammatory signaling pathways [18,19,20]. These alterations can impact immune surveillance and responses to immunotherapy, highlighting the need to further investigate how CIN-driven immune remodeling affects tumor progression and treatment outcomes.

This review explores the mechanisms underlying CIN and CH in BC and their clinical implications. We discuss how CIN and CH contribute to tumor evolution, treatment resistance, and disease progression. Additionally, we highlight the role of advanced detection methods, such as single-cell sequencing, in characterizing these phenomena. Understanding the molecular mechanisms associated with CIN and CH could lead to the identification of novel biomarkers, improving diagnostic, prognostic, and therapeutic strategies in BC and advancing precision medicine approaches.

## 2. CIN and CH: Cellular Mechanisms and Their Implication for Cancer

CIN refers to a phenomenon in which cells exhibit elevated rates of chromosomal alterations, including the loss, gain, or rearrangement of chromosomes during cell division [14]. This instability is a key factor in cancer progression, fostering genetic diversity within tumors and enabling them to adapt to various stressors, such as cytotoxic anticancer treatments.

CIN can be classified into two main types: numerical and structural. Numerical CIN involves the gain or loss of entire chromosomes (aneuploidy), which is an inevitable consequence of CIN. In contrast, structural CIN involves alterations in the structure of chromosomes, such as deletions, duplications, the translocations of chromosomal segments, and other alterations [21].

Numerical and structural CINs are crucial in tumorigenesis and the development of resistance to treatment [22]. Many of these numerical and structural chromosomal alterations are non-clonal chromosomal abnormalities (NCCAs), and since these changes are not clonal (unlike clonal chromosomal alterations (CCAs)), they are often overlooked and, as a result, not reported [23]. CCAs are defined as chromosomal alterations observed at least twice in 20 to 40 randomly examined mitotic figures, with an occurrence frequency greater than 30% [24]. In contrast, NCCAs are defined as non-recurrent chromosomal alterations observed at a frequency of less than 4% among 50–100 mitotic figures [24] and are characteristic of chaotic genomes. Since CCAs are indicative of stable karyotypes and NCCAs are associated with unstable karyotypes, it has been suggested that NCCAs are key indicators of structural CIN and cancer evolution [25].

Additionally, CIN has been used to describe the presence of high-level aneuploidy in tumors with complex karyotypes. Consequently, the “CIN phenotype” broadly encompasses alterations in chromosome copy number, ploidy, and structural modifications [21].

Given that the specific type of CIN is often not emphasized in the literature, this review defines CIN as a result of defects in the mechanisms that drive the generation of new cytogenetically distinct clones and aneuploid subclones at a rate exceeding that of normal cells.

### 2.1. Cellular Mechanism of CIN

**Figure 1:**
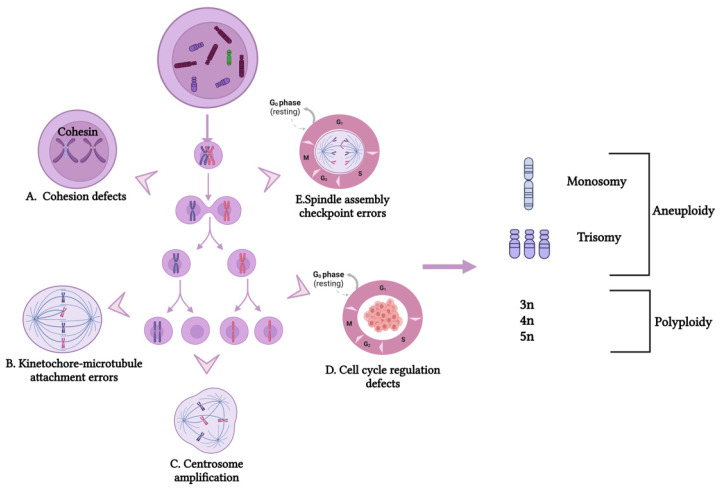
Cellular mechanisms leading to numerical chromosomal instability (CIN) in cancer. Numerical CIN arises from errors in chromosome segregation during mitosis (mitotic nondisjunction), leading to daughter cells with aneuploid or polyploid karyotypes. Key mechanisms involved include (**A**) cohesion defects, where a loss of cohesin (blue) prevents proper chromatid alignment in metaphase, causing segregation errors in anaphase; (**B**) kinetochore–microtubule attachment errors, where incorrect spindle attachment results in sister chromatids migrating to the same pole, leading to missegregation; (**C**) centrosome amplification, where extra centrosomes increase erroneous kinetochore attachments and chromosome lagging; (**D**) cell cycle regulation defects, where checkpoint failures allow cells with missegregated chromosomes to continue division; and (**E**) spindle assembly checkpoint (SAC) errors, where improperly attached chromosomes should activate SAC to delay anaphase, but undetected errors lead to missegregation.

**Figure 2:**
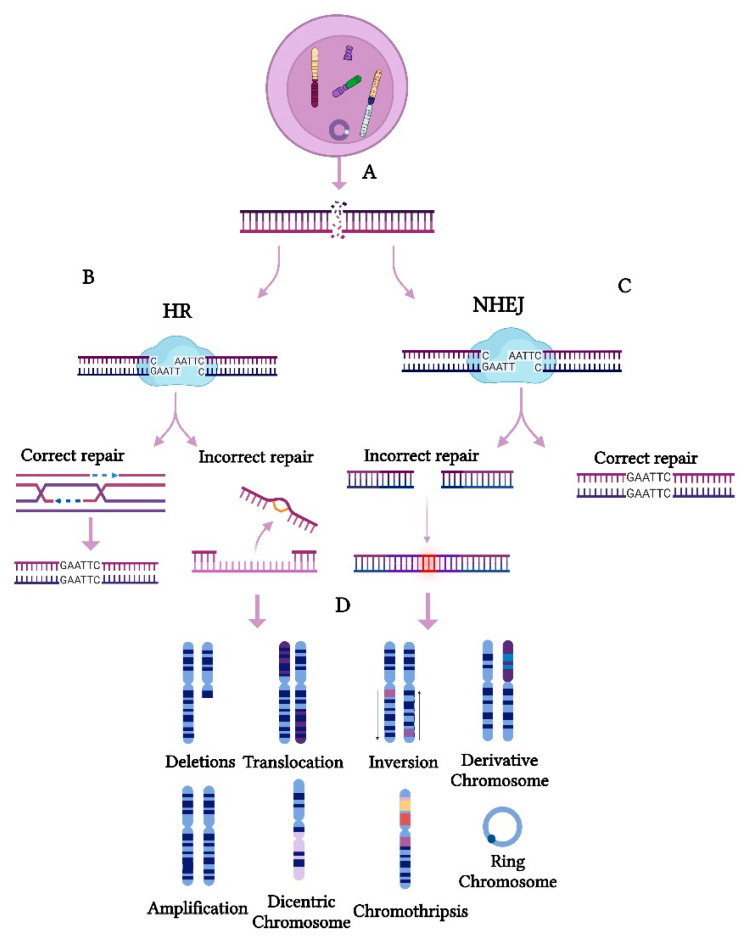
Cellular mechanisms leading to structural chromosomal instability (CIN) in cancer. Structural CIN results from deficiencies in genome integrity maintenance, primarily affecting (**A**) double-strand break (DSB) repair. (**B**) Homologous recombination (HR) ensures accurate DSB repair by using an undamaged homologous sequence as a template. HR defects lead to increased reliance on error-prone pathways. (**C**) Non-homologous end joining (NHEJ) leads to an increased reliance on error-prone pathways, as it joins DNA ends without the guidance of a homologous sequence. (**D**) When either of these mechanisms fails to function properly, the affected DNA strand ends remain exposed, potentially leading to structural chromosomal alterations such as deletions, translocations, inversions, derivatives chromosomes, amplifications, dicentric chromosomes, ring chromosomes, and chromothripsis. Several processes contribute to the formation of these chromosomal alterations, including splicing, resection, alignment, strand invasion, and/or replication, all of which promote the induction of CIN.

The mechanisms underlying CIN remain incompletely understood but are thought to involve errors in DNA replication and chromosome segregation during cell division. These errors result in cells with abnormal chromosome numbers (numerical CIN) and/or structures (structural CIN) (Figure 1 and Figure 2). Numerical CIN arises from defects in mechanisms that ensure proper sister chromatid segregation [26,27]. These defects include cohesion defects (Figure 1A), kinetochore–microtubule attachment errors (Figure 1B), centrosome amplification (Figure 1C), cell cycle regulation defects (Figure 1D), and spindle assembly checkpoint (SAC) errors (Figure 1E). Such errors lead to the gain or loss of entire chromosomes, contributing to aneuploidy and genomic instability in cancer cells [28].

In contrast, structural CIN arises from defects in genome integrity maintenance mechanisms, including double-strand break (DSB) repair (Figure 2), replication stress management, and non-allelic homologous recombination [29]. DSBs are among the most severe types of DNA damage (Figure 2A) and, if not correctly repaired, can lead to structural CIN in cancer. Homologous recombination (HR) is the most precise repair pathway for DSBs, playing a crucial role in preserving genomic stability (Figure 2B).

However, defects or dysregulation in HR can compromise this repair process, increasing the reliance on error-prone pathways such as non-homologous end joining (NHEJ) (Figure 2C), which contribute to chromosomal amplifications, deletions, translocations, the formation of extrachromosomal structures, dicentric chromosomes, ring chromosomes, chromothripsis, and complex large-scale rearrangements [27,30,31] (Figure 2D). Furthermore, impaired HR repair can exacerbate replication stress, leading to stalled replication forks and additional DNA breakage, further driving chromosomal rearrangements [32]. These genomic alterations enhance tumor heterogeneity, promote cancer progression, and contribute to overall CIN and tumor evolution [1]. Notably, numerical and structural CINs often coexist in tumor cells, creating a complex interplay that fuels cancer progression [33,34].

### 2.2. CIN in Cancer Progression

**Figure 3:**
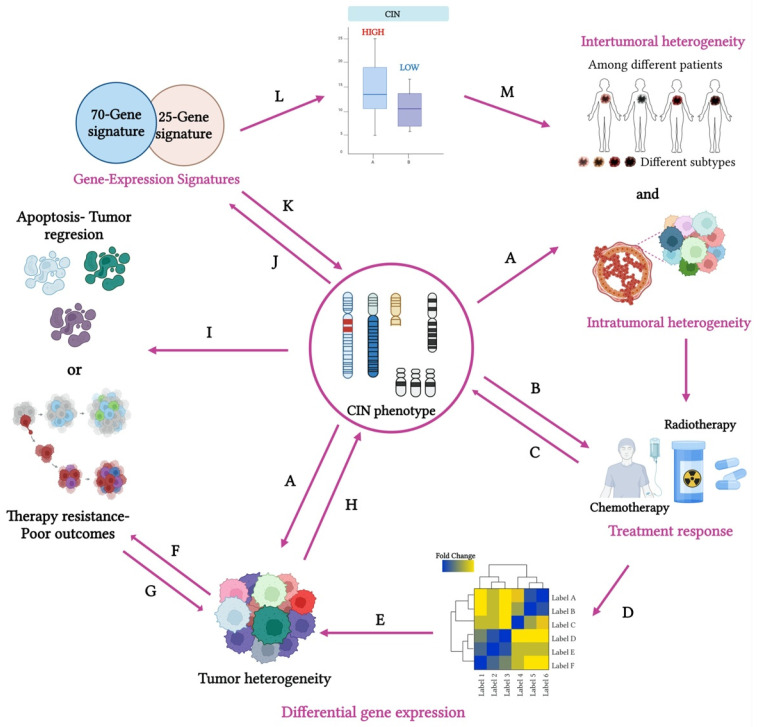
The role of the CIN phenotype in breast cancer progression, therapy resistance, and patient outcomes. CIN plays a crucial role in tumor evolution and progression, contributing to both (**A**) intertumoral and intratumoral heterogeneity. The administration of chemotherapy or radiotherapy to tumor cells (**B**) can increase CIN (**C**) and alter gene expression (**D**), both of which are associated with a rise in tumor heterogeneity (**E**). This increased heterogeneity can lead to the induction of apoptosis (**F**), resulting in tumor regression, or the clonal expansion of new oncogenic alterations, further enhancing heterogeneity (**G**) and CIN (**H**) and driving resistance to therapy (**I**). Given the strong association between CIN and variations in gene expression (**J**), signatures such as CIN25 and CIN70 have been investigated as potential biomarkers to evaluate CIN (**K**) and stratify BC patients based on their risk (**L**). These signatures hold the potential to improve the accuracy of diagnostic predictions and patient outcome assessments (**M**).

Genomic analyses have shown that chromosome-arm somatic copy-number aberrations occur more frequently than whole-chromosome alterations. Moreover, specific chromosomal arms are preferentially lost or gained, indicating that these events are selectively favored due to their advantageous role in cancer progression [35,36]. In this context, CIN plays a crucial role in cellular transformation, tumor evolution, and disease progression, driving both intertumoral and intratumoral heterogeneity [37,38] (Figure 3A,B). This feature is strongly associated with metastasis and the development of drug resistance [39]. Moreover, by increasing genetic and phenotypic heterogeneity within tumors, CIN not only accelerates tumor progression but also significantly influences treatment outcomes [40,41]. As a result, the genetic diversity generated by CIN allows cancer cells to adapt to fluctuating conditions, including the selective pressures imposed by treatments, ultimately promoting tumor progression [42].

In BC, CIN has been strongly associated with poor prognosis, as it drives rapid and dynamic tumor evolution, promoting metastasis and increasing the risk of recurrence [43]. According to a recent study, this evolutionary process follows distinct genomic pathways that shape tumor progression, immune interactions, and treatment responses. Specifically, a meta-cohort analysis of 1828 breast tumors, spanning the pre-invasive, primary invasive, and metastatic stages, identified three distinct genomic archetypes. ER-positive (ER+) high-risk tumors exhibit complex focal amplifications, similar to HER2+ tumors, including cyclic extrachromosomal DNA amplifications that arise in pre-invasive lesions. In contrast, triple-negative tumors display genome-wide instability, tandem duplications, and homologous recombination deficiency-like signatures, whereas ER+ typical-risk tumors remain largely genomically stable [44]. These genomic archetypes play a crucial role in BC progression, as they emerge early in tumorigenesis, actively shape the tumor microenvironment, and persist throughout metastatic disease. Their stability across different stages of cancer suggests that they may drive tumor evolution and influence disease outcomes.

In line with this, a recent study characterized the evolution and maintenance of chromosome 4p (chr4p) loss in basal BC, identifying it as a recurrent deletion in this subtype [45]. The phylogenetic analysis of primary tumor/patient-derived xenograft basal breast cancers demonstrated that chr4p deletion emerges early in tumor evolution. Functionally, this loss was associated with enhanced proliferation. Further gene function studies identified *C4orf19*, a previously uncharacterized gene within chr4p, as a key regulator of proliferation suppression when overexpressed. Additionally, it was indicated that certain chromosomal regions, including chr4p, suppress proliferation in a context-dependent manner, highlighting network-level interactions. These findings underscore the early emergence of complex aneuploid karyotypes in basal BC, particularly involving chr4p, and reveal adaptive genomic landscapes that drive tumor progression.

Building on these insights, an integrated genome and transcriptome analysis of a large breast tumor cohort revealed that CIN, driven by inherited variants (CNVs and SNPs) and somatic copy number aberrations (CNAs), affects gene expression in 40% of genes, mainly through cis- and trans-acting CNAs [46]. Furthermore, key cancer-related deletions in *PPP2R2A*, *MTAP*, and *MAP2K4* highlight CIN’s role in tumor progression. In addition, unsupervised clustering of DNA–RNA profiles identified two subgroups with low copy numbers and cis-acting alterations but distinct genomic instability levels and clinical outcomes. Specifically, one subgroup, characterized by low genomic instability, was mainly composed of luminal A cases and histotypes associated with a good prognosis, such as invasive lobular and tubular carcinomas. Meanwhile, the second subgroup, termed the “CNA-devoid” subgroup, included both ER-positive and ER-negative cases with a flat copy number landscape. Moreover, the analysis revealed that most basal-like tumors formed a stable subgroup with high genomic instability. Notably, this subgroup exhibited distinct cis-acting alterations, including 5q loss and gains in 8q, 10p, and 12p. Despite their high genomic instability, these tumors showed relatively good long-term outcomes [46]. These findings underscore the impact of CIN on breast cancer stratification and progression.

Notably, recent studies indicate that CIN is present in 89% of invasive BC patients, underscoring its potential relevance for both diagnosis and treatment [11]. Gaining a deeper understanding of the mechanisms underlying CIN in BC is crucial for developing targeted therapies to address tumor heterogeneity and improve patient outcomes.

### 2.3. Polyploid Giant Cancer Cells (PGCCs) and CIN

PGCCs represent a distinct subpopulation of tumor cells, characterized by their abnormally large size and increased DNA content resulting from whole-genome duplication. These cells arise through endoreplication and are considered a specialized subpopulation contributing to solid tumor heterogeneity [47].

PGCCs arise in response to various stressors, including hypoxia, chemotherapy, and radiation, enabling tumor survival under adverse conditions [48]. Among the adaptive mechanisms of PGCCs, the development of polyploidy plays a crucial role by facilitating the evasion of senescence in chemotherapy-treated cancer cells, further driving tumor progression [49]. Furthermore, PGCCs have been linked to key oncogenic processes, including immortalization, transformation, and RAS-mediated tumor initiation and metastasis [50]. In this context, tetraploid cells, rather than diploid cells, have been identified as primary drivers of tumorigenesis [51]. Consistently, a strong association between PGCCs and CIN has been established, as the polyploid nature of these cells leads to abnormal mitotic divisions, driving extensive genomic heterogeneity [52]. This heterogeneity fuels clonal evolution, promoting tumor progression, metastasis, and resistance to therapy [47]. Additionally, PGCCs can undergo depolyploidization, giving rise to highly adaptable and aggressive progeny with enhanced tumorigenic potential [47].

Therefore, PGCCs represent a key driver of CIN-associated tumor evolution, playing a pivotal role in cancer aggressiveness and therapeutic resistance [53]. In BC and other malignancies, PGCCs arise in response to therapy-induced stress, generating progeny with cancer stem cell properties capable of repopulating the tumor. By influencing the tumor microenvironment, PGCCs drive BC progression, enhance chemoresistance, and promote metastasis and relapse, ultimately affecting patient survival [54].

Given their pro-tumorigenic functions, PGCCs have been proposed as potential predictors of treatment responses and prognosis.

### 2.4. Karyotype Coding: The Two-Phased Cancer Evolution Model and CIN

Since CIN drives cancer evolution through large-scale chromosomal alterations that fuel both macroevolutionary shifts and subsequent microevolutionary adaptations, deciphering these processes is essential for identifying strategies to control tumor progression and therapeutic resistance. Macroevolutionary shifts refer to major genomic rearrangements, such as whole-genome duplications, chromosomal gains or losses, and structural variations that can create new tumor subclones with distinct phenotypic traits [9]. These large-scale changes can drive rapid tumor evolution, leading to the emergence of aggressive clones with enhanced survival capabilities [55]. In contrast, microevolutionary adaptations involve more gradual, stepwise genetic alterations within subclonal populations, such as point mutations, copy number variations, and epigenetic modifications [5]. These fine-tuned adaptations allow cancer cells to respond to selective pressures, including immune surveillance and therapeutic interventions, ultimately contributing to tumor heterogeneity and resistance [8].

In this context, the Cancer Genome Project identified CIN as a pivotal driver of cancer evolution, as predicted by the two-phased cancer evolution model [56]. This model provides a novel conceptual framework for cancer research, emphasizing that chromosomal alterations initiate macroevolution, which subsequently gives rise to microevolution through gene mutations and epigenetic modifications. These findings reinforce the concepts of karyotype coding and cancer evolution, underscoring CIN’s crucial role in tumor adaptation and malignancy.

In line with this, a recent study proposed a general mechanism for two-phased cancer evolution, wherein genome alteration-driven macroevolution precedes gene mutation- and epigenetic alteration-driven microevolution [57]. In general terms, this means that under high-stress conditions, genome chaos triggers macroevolution, ensuring the survival of new cellular systems, while microevolution facilitates system modifications, promoting proliferation and competition [57]. This dynamic interplay between macroevolution and microevolution underscores the complexity of tumor progression and highlights potential therapeutic targets to mitigate cancer aggressiveness and resistance.

This model has been extended to major phase transitions in cancer evolution [9], including the progression from a localized tumor to metastasis and from drug sensitivity to resistance [55,58,59,60]. Each transition involves multiple NCCA/CCA cycles throughout cancer progression, from benign growth to metastasis and drug resistance [8,61].

Notably, in BC, the existence of punctuated or discontinuous cancer evolution has been confirmed through single-cell sequencing [62].

Collectively, these studies strengthen the concepts of karyotype coding and cancer evolution, highlighting the pivotal role of CIN in tumor adaptation and malignancy.

### 2.5. CH in Cancer Progression

CH plays a crucial role in the complexity of cancer, as it reflects the coexistence of distinct subclones within a tumor. Each subclone may possess unique genomic, epigenomic, transcriptomic, and proteomic features that contribute to differences in tumor characteristics, such as proliferation, apoptosis, metastasis, and response to therapy. This clonal diversity is observed both between tumors from different patients or metastatic sites (intertumoral heterogeneity) and within the same tumor in an individual patient (intratumor heterogeneity) (Figure 3A), creating a dynamic environment where subclones may compete or cooperate [11]. This diversity could have significant implications for disease progression, treatment responses, and overall cancer management (Figure 3B). In particular, CH is associated with the presence of multiple subclones, each potentially exhibiting distinct responses to therapeutic agents. This variability could drive an increase in CIN (Figure 3C) and alter gene expression patterns (Figure 3D), both of which contribute to increased tumor heterogeneity (Figure 3E). This increased heterogeneity can lead to two distinct cellular outcomes. First, it can induce apoptosis (Figure 3F), likely triggered by excessive genotoxic stress. This process results in tumor regression and an improved prognosis for the patient. Alternatively, it can promote the clonal expansion of new oncogenic alterations (Figure 3F). These alterations further amplify heterogeneity (Figure 3G) and increase CIN (Figure 3H), ultimately driving resistance to therapy (Figure 3I).

In turn, aneuploidy, a direct consequence of CIN, is closely linked to variations in gene expression (Figure 3J), offering valuable insights into the molecular mechanisms driving CIN [63]. To further explore these mechanisms, several gene expression-based CIN panels have been specifically developed for studying BC [64] (Figure 3K).

Building on these insights, recent advances in DNA sequencing technologies have allowed for a detailed tracing of CH and tumor evolution, uncovering its underlying genomic alterations. These techniques have demonstrated that early driver mutations and alterations in cancer-related genes generate genetic diversity, shift selective pressures, and facilitate the expansion of distinct subclones [65,66].

In line with this, the complex CH within BC tumors has also been demonstrated using DNA barcoding and high-throughput sequencing of samples from xenograft transplantation mouse models of human BC [67,68]. These studies revealed that while some subclones remain stable, others either expand or disappear over time, further confirming the dynamic nature of CH. As a result, BC progression is marked by significant changes in the number and size of subclones, as well as the emergence of new ones, both within and between primary and secondary tumors. Therefore, CH plays a crucial role in shaping the dynamic evolution of BC. As BC progresses, distinct subclones may develop unique mutations, resulting in diverse behaviors such as therapy resistance or enhanced metastatic potential.

### 2.6. The Impact of CIN and CH on Therapeutic Resistance and Patient Outcomes

CIN is a predictor of drug resistance and poor prognosis across various cancer types [69,70]. Several recent studies have highlighted the role of CIN, CH, and clonal evolution in driving chemotherapy resistance and metastasis [69]. By enabling the development of diverse subclones within the tumor, CIN contributes to therapeutic resistance, disease recurrence, and metastasis, making it a critical factor in the aggressiveness and poor prognosis of various cancers. This relationship may be explained because CIN not only promotes tumor evolution but also complicates treatment by diminishing the efficacy of targeted therapies, underscoring the necessity of dynamic, personalized approaches to manage cancer patients effectively.

Importantly, it has been described that CIN-mediated genetic and phenotypic diversity in tumors has significant implications for chemotherapy resistance through three potential pathways: innate resistance, acquired resistance, and adaptive resistance [69]. Innate resistance, defined as a pre-existing insensitivity of cancer cells to therapy due to intrinsic cellular factors, is possibly related to the need for cancer cells to tolerate CIN and aneuploidy, which may create a higher threshold for tolerance to chemotherapy agents [71]. Innate resistance, possibly related to the need for cancer cells to tolerate CIN and aneuploidy, may create a higher threshold for tolerance to chemotherapy agents.

Additionally, it is possible that the aneuploidy-mediated reduction in growth rate observed in some models leads to a quiescent state, offering protection against DNA-damaging agents or other chemotherapeutics. Acquired resistance, resulting from the high diversity of the tumor cell population before chemotherapy, increases the likelihood that a subclone with intrinsic protection against the chemotherapy agent exists. These subclones can then outgrow the sensitive tumor cells that are eliminated during treatment [13]. Adaptive resistance occurs when a resistant subclone is not present before treatment but emerges during or after chemotherapy. This may depend on a mutator phenotype that creates genetic diversity through CIN or small-scale changes like point mutations. This mutator ability could be intrinsic to the tumor (e.g., tumor CIN) or enhanced by the genome-destabilizing effects of cytotoxic chemotherapy agents [13,63]. In fact, CIN and its associated aneuploidy have been shown to predict responses to both chemotherapeutic and immunotherapeutic treatments [13,70]. Furthermore, CIN has demonstrated potential as a predictive marker for patients’ response to specific therapies, offering opportunities to enhance personalized medicine strategies.

Notably, in a landmark study, researchers identified a gene expression signature that correlated with poor clinical outcomes and metastasis across multiple cancer types [72]. Given the potential limitations of this study, principally related to cell proliferation as a confounding factor, another subsequent work proposed an alternative signature of CIN, supporting its relationship with poor clinical outcomes [73]. More recently, large-scale and high-throughput studies have explored in more detail the mechanistic basis of such associations and found potential biomarkers for the prognosis and cancer therapy response [43,70,74]. Another intriguing finding, validated in multiple cancer types, is that extremely high CIN is correlated with a more favorable prognosis rather than worse prognostic outcomes [12,69].

In gastric cancer, for instance, a recent study investigating the predictive and prognostic value of CIN and other biomarkers demonstrated that patients with high CIN had better outcomes when treated with neoadjuvant chemotherapy compared to those with low or moderate CIN [75].

Similarly, breast cancers with extreme CIN are thought to have a better prognosis than those with intermediate CIN, potentially due to their heightened sensitivity to adjuvant chemotherapy [69].

From an evolutionary and ecological perspective, these observations could be attributed to the reduced fitness of cancer cells with highly unstable chromosomes and high CH, a hypothesis referred to as the “just-right” model (Figure 3F) [75]. On one hand, cancer cells with low CIN exhibit limited adaptability to environmental conditions. On the other hand, cells with extremely high CIN levels surpass the threshold of viability and may succumb to cell death [13]. In contrast, cells with moderate “just-right” levels of CIN demonstrate greater adaptability to the dynamic conditions of the tumor microenvironment, enabling them to survive, replicate, and proliferate. This paradoxical effect, often associated with aneuploidy, reflects the complex panorama of CIN in cancer development, progression, and control [33,75,76,77]. These insights offer important clinical implications that could help refine treatment strategies and enhance patient outcomes.

## 3. CIN Phenotype and CH: Detection Methods

Although CIN and CH constitute important tumor features, they are rarely measured in clinical practice due to technical challenges in their assessment and current limited applications [78,79]. Several techniques have been proposed to quantitatively assess the CIN phenotype. These methods along with their advantages and limitations are presented in Table 1.

In this context, a recent study evaluated various techniques to estimate CIN, including conventional methods such as fixed imaging, karyotyping, fluorescent in situ hybridization (FISH), bulk RNA-seq, and single-cell DNA sequencing [79]. The results of this study showed that conventional methods including fixed imaging, karyotyping, and FISH required many proliferative mitotic cells, which are not easily obtained in some clinical specimens. Bulk RNA sequencing, although appealing due to its availability and efficiency, failed to detect CIN in any instability models. Conversely, single-cell DNA sequencing revealed a higher correlation with CIN scores in MCF10A cells, suggesting that single-cell sequencing is a sensitive technique to measure CIN. Furthermore, multiple lines of evidence suggest that the clinical relevance of CIN and aneuploidy as prognosis biomarkers and therapeutic targets is context-dependent and cancer type-specific [79].

Notably, when comparing these methods, some techniques demonstrate greater accuracy or versatility depending on the type of sample. However, there is currently no universally accepted “gold standard” for measuring the CIN phenotype. Further studies are needed to compare these techniques and to explore new approaches for assessing the CIN phenotype, such as utilizing gene expression signatures, scDNA-seq, or scRNA-seq in clinical samples. These advanced methods provide valuable insights into the molecular changes associated with CIN, including alterations in gene regulation and key cellular processes such as cell cycle control and DNA repair. By integrating these techniques, researchers can achieve a more comprehensive understanding of CIN-driven cancer evolution and its impact on patient prognosis and treatment responses.

### 3.1. Gene Expression Signatures as Indicators of the CIN Phenotype and CH

The use of gene expression signatures to evaluate the CIN phenotype captures the dynamic changes in cellular gene regulation that arise in response to varying levels of CIN. These signatures are not static; rather, they reflect the influence of CIN on cellular processes such as adaptation, survival, and stress response, all of which contribute to the progression of cancer. For example, in cases of moderate CIN, gene expression patterns have been linked to increased adaptability, as CIN introduces genetic variability that enables cancer cells to evolve and gain survival advantages. This aligns with the “CIN paradox”, where CIN paradoxically promotes tumor progression by generating gene expression profiles that enhance survival and growth [80].

On the other hand, at high CIN levels, the gene expression signatures often reflect a breakdown in cellular homeostasis. The genomic instability produced by high CIN levels can cause catastrophic chromosomal aberrations, triggering stress response pathways and resulting in the activation of apoptosis or senescence [81]. These alterations suggest that gene expression signatures at high CIN levels could reflect not only the presence of CH and aneuploidy but also the extent to which the cellular environment is destabilized.

Gene expression signatures are closely linked to CIN and aneuploidy, offering valuable insights into the ongoing processes of CIN and the potential for adaptation in cancer cells [82,83]. These signatures provide a deeper understanding of the CIN phenotype. Building on this, the identification of specific gene signatures that predict the CIN phenotype has garnered significant attention in BC research due to its potential clinical implications.

Identifying gene signatures that accurately reflect CIN levels can therefore provide valuable insights for predicting tumor behavior and patient outcomes. Since aneuploidy results from CIN, genes whose expression levels are consistently linked to aneuploidy could offer valuable insights into the molecular mechanisms driving CIN (Figure 3J). In fact, a strong correlation has been identified between alterations in chromosome copy number and variations in gene expression in the affected regions [69,84].

Notably, gene sets associated with mitotic spindle assembly, the spindle checkpoint, and the DNA damage checkpoint have shown significantly higher CIN scores compared to those involved in the cell cycle [84].

These observations support the hypothesis that the overexpression of these specific cell cycle control genes is linked to CIN, rather than simply an increased proportion of mitotic cells. In contrast, gene sets related to DNA repair, centrosome duplication, and chromosome segregation were not significantly enriched in CIN scores relative to cell cycle-regulated genes. Our knowledge about gene expression signatures is evolving rapidly, and their development is expected to provide deeper insights into the intrinsic features of tumors. This progress not only promises to improve our understanding of cancer biology but also to optimize treatment strategies in clinical practice.

Several gene expression-based CIN panels have been developed to study various types of cancer. In particular, gene signatures such as CIN25 and CIN70 have been explored as biomarkers to assess CIN and to stratify patients based on their risk (Figure 3K) [72]. Designed to identify tumors with unstable genomes, these signatures also predict the potential for metastasis. Their ability to capture underlying genomic instability could enable more accurate predictions of diagnosis and patient outcomes, potentially surpassing traditional histopathological methods. This underscores their clinical relevance in risk stratification (Figure 3L) and personalized cancer treatment (Figure 3M). Additionally, both signatures have been associated with a poor prognosis, a higher tumor grade, and resistance to therapy.

In addition to the previously presented CIN25 and CIN70 scores, a panel of 17 marker genes associated with genomic instability in breast tumors was also identified, further supporting the use of expression signatures to improve the prediction of clinical outcomes in BC patients [73].

#### The Role of CIN25 and CIN70 Gene Signatures in Predicting CIN and Their Clinical Implications

The CIN25 signature is a more compact set of 25 genes that similarly reflect processes linked to CIN, such as mitotic errors, DNA damage responses, and cell division regulation. This signature has been used to assess the association between CIN and tumor aggressiveness. In contrast, the CIN70 signature includes a set of 70 genes related to cell cycle progression, DNA repair, the spindle assembly checkpoint, DNA replication, and chromosome segregation. Both signatures have been investigated as biomarkers for CIN and metastatic potential across different cancer types [72]. These studies revealed that metastatic samples exhibited higher expression levels of the CIN25 signature compared to primary tumors, with a similar pattern observed for the CIN70 signature. Moreover, a subset of primary tumors showed elevated relative expression of these signatures, supporting the hypothesis that the metastatic expression profile originates in the primary tumor and remains consistent in distant metastases.

In BC, both the CIN25 and CIN70 signatures have shown significant potential to improve tumor classification and predict clinical outcomes by reflecting the genomic instability associated with more aggressive subtypes. For example, studies have explored the relationship between the CIN25 gene signature and the tumor grade across various breast cancer datasets. These analyses revealed that within grade 1 and grade 2 tumors, higher CIN25 expression was linked to poorer clinical outcomes. However, grade 3 tumors consistently exhibited elevated CIN25 expression, making it difficult to distinguish them based solely on this measure [72]. Similarly, some studies have shown that the CIN70 signature correlates more strongly with the tumor proliferation rate than with intrinsic CIN. Rather than accurately predicting CIN, the CIN70 score primarily reflects cell proliferation dynamics [82]. Likewise, recent studies have shown that CIN70 does not correlate with the microscopic detection of mitotic defects, suggesting that it is an unreliable marker of ongoing CIN [79]. This highlights the challenges of using gene expression signatures for stratification in advanced-grade tumors, as their predictive value may vary depending on the biological processes they primarily reflect.

Given the high heterogeneity of BC, continued research is essential to more effectively stratify tumor subtypes based on their CIN phenotype, thereby improving diagnosis and the prognosis. Although these measures have rarely been applied in clinical diagnostics due to technical challenges and unclear significance, their broader use could significantly enhance our understanding of tumor biology and improve patient stratification and treatment outcomes.

### 3.2. Single-Cell RNA Sequencing in the Study of the CIN Phenotype and CH

Although the CIN25 and CIN70 gene signatures have provided a valuable means to assess the potential role of the CIN phenotype in determining the malignant potential of tumors, their evaluation is based on the analysis of genetic material from a pooled population of cells, rather than on a cell-by-cell basis. This approach may overlook important CH within the tumor, as individual cells can exhibit varying degrees of CIN. As a result, pooled analyses might mask the presence of subpopulations of cells with different genomic profiles, potentially affecting the accuracy of the CIN assessment and its correlation with clinical outcomes. Considering these limitations, single-cell sequencing techniques emerge as valuable tools for evaluating both CIN and CH. This strategy could enable the precise identification of distinct cellular subpopulations, potentially enhancing our understanding of tumor diversity and improving the accuracy of CIN assessments in relation to patient prognosis.

However, added to this complexity is the paradoxical relationship between CIN status and clinical outcomes, which poses a dual challenge: identifying this specific CIN pattern and defining thresholds for accurate prognostic prediction. Furthermore, given the potential variability in CIN levels, a clinically relevant test must establish clear thresholds to enable oncologists to differentiate between patients with a favorable or unfavorable prognosis. Determining these thresholds is particularly challenging, as the relationship between CIN levels and clinical outcomes is likely intricate. Addressing these challenges, along with advancing and clinically applying innovative methods to detect and evaluate CIN, is crucial for the effective integration of precision medicine tools in the future.

Despite the importance of cellular diversity arising from CIN and CH in tumor progression, relapse, and therapy resistance, robust quantitative methods to assess such traits remain elusive [85]. Recently, the advent of new technologies such as scRNA-seq has enabled the direct and individual assessment of the CIN phenotype and CH with potential research and clinical applications [86].

#### 3.2.1. Overview of scRNA-seq Technology and Its Relevance to Cancer Research

scRNA-seq is a robust method in transcriptomics that allows for high-throughput and high-resolution analysis by isolating individual cells and generating RNA sequencing libraries, followed by massively parallel sequencing [86]. This approach has provided an unprecedented opportunity to unravel cell-to-cell variability, providing valuable insights into biologically relevant differences between cells, cell populations, and their transcriptomic dynamics [87].

In tumor biology, the advent of these new technologies has led to significant advances in our understanding of cancer initiation and progression, particularly in areas such as CH, the tumor microenvironment, invasion, metastasis, relapse, and therapy response [88]. In addition, a growing number of studies using these technologies have highlighted their potential value in clinical settings. For instance, it was demonstrated that CIN is responsible for gene expression changes and contributes to cell heterogeneity among glioblastoma cancer stem cells, thereby contributing to therapy resistance and posing a significant challenge to cancer treatment [89]. More recently, the role of intratumoral B cells and their association with several tumor traits has been studied in BC and other solid tumors [90]. Interestingly, the authors identified subsets of B cells potentially linked to the immune checkpoint inhibition response through mechanisms that maintain genomic stability and drive mutagenesis, thus opening new avenues for research and therapeutic strategies. In this context, recent studies have also highlighted the crucial role of cancer cell-intrinsic inflammatory signaling driven by CIN. Specifically, it has been shown that cancers exhibiting CIN can evade the CIN-induced inflammatory response and, consequently, immune surveillance [18,19,20]. This suggests that tumors with CIN have evolved sophisticated mechanisms to modulate or exploit CIN-induced inflammation in a pro-tumorigenic manner.

The application of these technologies across multiple studies has dramatically enhanced our understanding of diverse cell types, gene regulatory network mechanisms, and transcriptional dynamics, detecting subtle but biologically significant differences. These findings deepen our understanding of tumor biology, providing new directions for cancer diagnosis, prognosis, and treatment.

#### 3.2.2. Applications of scRNA-seq in Mapping the CIN Phenotype and CH

**Figure 4:**
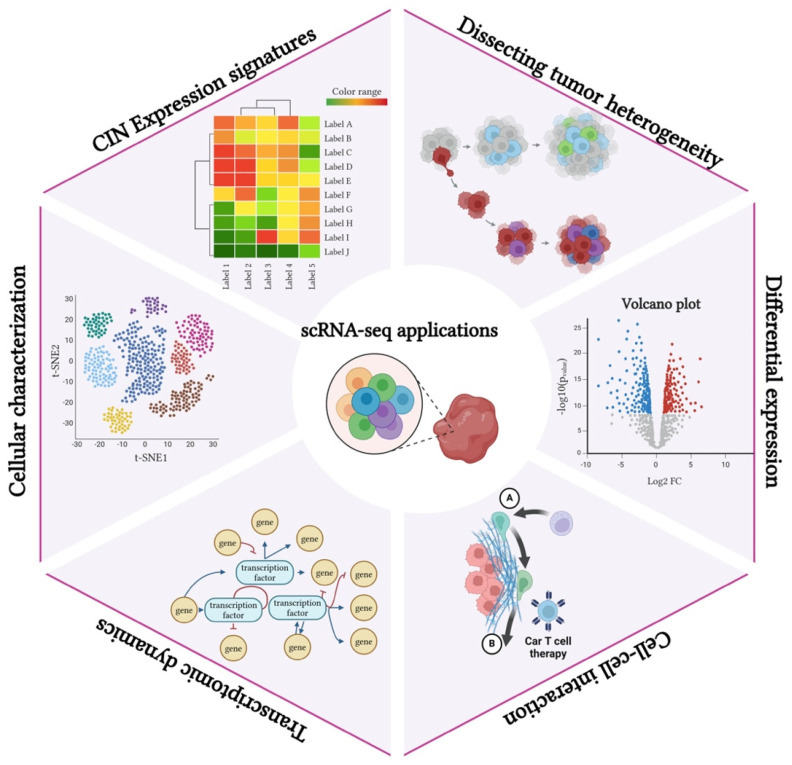
Application of scRNA-seq in tumor biology. scRNA-seq enables the identification of CIN expression signatures at the single-cell level, uncovering tumor heterogeneity and key molecular mechanisms. It facilitates differential expression analysis, cell–cell interaction characterization, and transcriptomic dynamics tracking, providing a high-resolution view of CIN in cancer development.

scRNA-seq has emerged as a powerful tool for assessing tumor biology and CIN. By capturing CIN expression signatures at the single-cell level, scRNA-seq enables the dissection of tumor heterogeneity, revealing distinct subpopulations within the tumor. This approach facilitates differential expression analysis, identifying key genes and pathways associated with CIN-driven tumor progression [91]. Moreover, scRNA-seq provides insights into cell–cell interactions within the tumor microenvironment, shedding light on how CIN influences communication between malignant and stromal cells [92]. Additionally, it allows for the study of transcriptomic dynamics, tracking changes in gene expression during tumor evolution [93]. Overall, scRNA-seq enhances cellular characterization, offering a high-resolution perspective on the molecular mechanisms underlying CIN and its role in cancer development (Figure 4).

#### 3.2.3. Insights Gained from Single-Cell Analyses Regarding the CIN Phenotype and Tumor Diversity

In recent years, scRNA-seq has been widely used to study tumor heterogeneity, the tumor microenvironment, CIN via transcriptional profiling, epigenetic regulation, and the discovery of new cell-specific and cell-type-specific markers [94]. The application of scRNA-seq has revealed both intratumoral and intertumoral heterogeneity, which are associated with tumor progression and patient outcomes. By analyzing the expression profiles of various cell types, including immune cells, scRNA-seq helps uncover mechanisms of immune evasion and drug resistance. Importantly, studies using this approach have shown that not only malignant cells but also other cells within the tumor microenvironment could be potential targets for precision oncology [95]. Also, scRNA-seq analyses have enabled the in-depth characterization of the tumor microenvironment, highlighting cellular diversity, cell–cell interactions, and the role of each cell type in tumor progression and prognosis [95]. Furthermore, the role of CIN has been associated with profound changes in tumor growth dynamics and cancer fitness at the single-cell level resolution [89].

In BC, these techniques have characterized various signatures associated with a poor prognosis and therapeutic resistance. For example, the analysis of a BC cell ecosystem revealed heterotopic interactions and the immunophenotype of diverse cell types. This approach allowed for tumor stratification into new groups called “ecotypes”, featuring unique cellular compositions and clinical outcomes, and provided a comprehensive high-resolution transcriptional atlas of the BC architecture. Furthermore, the investigation of the impact of CIN at the single-cell level has highlighted the complex relationship between this trait and disruptions in various signaling pathways [96]. For example, CIN induces chronic activation of the cGAS-STING pathway, promoting a pro-metastatic tumor microenvironment [97]. These studies underscore the importance of considering tumor heterogeneity, characterizing the tumor microenvironment, and understanding CIN related to cancer diagnosis, cancer prognosis, and therapeutic response.

## 4. CIN and CH Targeting and Therapeutic Potential

CIN, aneuploidy, and CH are widespread characteristics of cancer cells. It has been estimated that CIN can be found in above 70% of human cancers, being particularly prevalent in some of the most aggressive types of tumors, including triple-negative BC (TNBC), pancreatic cancer, lung cancer, and high-grade serous ovarian cancer [98,99,100,101]. Importantly, CIN, CH, and aneuploidy are inextricably intertwined and constitute attractive potential targets for prognosis, cancer treatment, and precision medicine. Despite the considerable progress in the understanding of these phenomena during the last decades, the translation of such findings in clinical settings remains challenging.

### 4.1. CIN and Aneuploidy as Therapeutic Targets

Considering the central role of CIN in carcinogenesis and tumor progression, a significant effort has been made to identify therapeutic targets. Initially described as “undruggable”, such efforts have led to important discoveries in cancer biology and the development of novel potential anticancer drugs [102,103]. Examples of these targets will be discussed below. In addition, sample evidence supports that CIN and CH are associated with resistance to commonly used anticancer therapies, including chemotherapy, radiotherapy, and immunotherapy [104,105,106].

### 4.2. Overview of Potential Therapeutic Strategies Targeting Aneuploidy, the CIN Phenotype, and CH

Aneuploidy, CIN, and CH have been topics of intense research in the last two decades [3,103]. Despite significant interest, there are currently no approved drugs specifically targeting CIN-related mechanisms. This can be attributed to several factors, including the diverse mechanisms driving CIN, which complicate the identification of specific molecular targets. Additionally, the technical challenges of measuring CIN in clinical samples, the dual role of CIN in both, tumor growth and progression (suggesting a narrow therapeutic window), the high genomic plasticity of tumor cells, and the complex interplay of CIN within the tumor microenvironment, particularly in relation to the immune response, further hinder therapeutic advancements [33,107].

Notably, several promising therapeutic strategies have been proposed, and clinical trials are ongoing to develop new anticancer drugs. CIN therapeutic strategies may be grouped according to different aspects and molecular characteristics of CIN [107]. Although, in some cases, these strategies are not completely independent, they exemplify the promises and pitfalls of targeting CIN in cancer.

One of these strategies is reducing CIN, which in turn could decrease tumor progression, adaptability, and resistance to current therapies. This may occur by restoring the functional activity of pathways that prevent CIN, for example, p53 and RB in unstable cancers. One example of this strategy is rezatapopt, a first-in-class p53 reactivator that selectively targets the p53 Y220C mutant protein, restoring its conformational and transcriptional activity [108]. Preliminary results from the PYNNACLE study, a multicenter phase 1/2 clinical trial evaluating the safety, efficacy, and side effects of rezatapopt in solid tumors, have shown promising tolerability and effectiveness in cancer patients [109]. Another approach to target p53 is by protecting it from negative regulators such as MDM2 and MDMX. This can be achieved by directly inhibiting MDM2, blocking the MDM2/p53 interaction, or using dual inhibitors that target both MDM2 and MDMX [110]. Ongoing clinical trials exploring these strategies may lead to the development of new therapeutic agents.

Conversely, another strategy involves increasing CIN, which may lead to a higher rate of chromosome missegregation and intolerable genomic alterations, ultimately inducing cell death. This approach could be explained by the “just-right”’ model described previously. Examples of this strategy are inhibitors of cell cycle checkpoints and critical processes during normal mitosis, including molecules that target aurora A, aurora B, and polo-like kinases. One of these targets is polo-like kinase 4 (PLK4), a serine–threonine kinase that plays a central role in centriole duplication [111]. The inhibition of PLK4 by CFI-400945, a selective small molecule, blocks centriole duplication causing increased aneuploidy, mitotic catastrophe, and cell death in different models [112,113]. Although promising, the main constraints of these inhibitors include limited efficacies and low tolerability in clinical trials [113,114]. An interesting approach to improve this strategy is identifying other molecular defects to leverage this vulnerability. A successful application of this approach in cancer treatment is synthetic lethality induced by poly (ADP-ribose) polymerase (PARP) inhibitors in tumors with homologous recombination repair defects (HRDs) [115]. Intriguingly, two recent studies highlight the role of TRIM37, a ubiquitin ligase required to prevent centriole reduplication, in the cancer-specific vulnerability to PLK4 inhibition [116,117]. Furthermore, TRIM37 is located in the 17q23 chromosomal region, a frequently gained region in BC including TNBC [45,118]. Altogether, these findings illustrate the potential of this strategy and point out the limitations and complexity of modulating CIN with therapeutic purposes.

Additional strategies involve targeting the effects of aneuploidy and CIN. Multiple studies have shown that CIN and aneuploidy trigger diverse cellular stress responses, including replication stress, proteotoxic stress, metabolic perturbations, mitotic disruptions, and immune alterations [81,119,120]. Two exciting and promising potential targets aimed at these vulnerabilities have been identified. The first is KIF18A, a member of the kinesin family of motor proteins required for maintaining mitotic spindle integrity. KIF18A is overexpressed in various cancers, including breast tumors, and has been associated with cell proliferation, cell invasion, and poor survival [121,122]. Interestingly, a recent study analyzed chromosomally stable and unstable cell lines, including those from TNBC, showing that the inhibition of KIF18A leads to significant mitotic delays and increased cell death [123]. Other studies have confirmed these observations, demonstrating that KIF18A inhibitors activate the spindle checkpoint, with cell death being highly selective for cancer cells exhibiting high CIN [124,125]. To date, at least three highly selective and potent KIF18A inhibitors have been identified, and clinical trials to assess their safety and efficacy are currently underway [126,127,128].

Another interesting research avenue is the modulation of the cGAS-STING pathway. Originally discovered as a sensor of viral DNA, this surveillance system regulates several tumor immunity responses [129,130]. This pathway is activated when endogenous or exogenous sources of cytoplasmic double-stranded DNA are sensed by the cyclic GMP-AMP synthase (cGAS), which catalyzes the conversion of GTP and ATP into the second messenger cyclic GMP–AMP (2′,3′-cyclic GMP-AMP—cGAMP) [129]. Subsequently, cGAMP binds and activates the adapter protein stimulator of interferon genes (STING). Finally, STING phosphorylates TBNK1, activating the type I interferon response. Notably, mitotic missegregation and chromosome fragments produced by genomic instability trigger the cGAS-STING pathway, promoting immunosurveillance and suppressing tumor progression [131,132]. Interestingly, a recent study showed that CIN triggers the IL-6/STAT3 signaling pathway located downstream of cGAS-STING, and the blockage of IL-6 signaling impairs tumor growth in a TNBC model with high CIN [133]. Furthermore, an additional study in BC patients found that the expression levels of cGAS-STING components are higher in TNBC and correlated with genomic instability and immune cell infiltration [134]. Paradoxically, cGAS/STING has also shown an oncogenic and metastatic potential, supporting the heterogenous role and complexity of this pathway and its modulation [135,136,137].

Finally, another potential strategy is targeting specific recurring aneuploidies products of CIN. Intriguingly, despite CIN having the potential to generate a vast number of chromosomal abnormalities, recurrent aneuploidies have been observed across specific tumors [138,139]. These observations may reflect positive selection and convergent evolution of similar chromosomal traits to increase tumor fitness [139,140]. In BC, for instance, a recent study analyzed a recurrent deletion on the short arm of chromosome 4 (chr4p) in the basal BC subtype, which is enriched in TNBC [45]. Similarly, the deletion on the short arm of chromosome 8 (chr8p) has been identified in a variety of human epithelial cancers, including BC [141]. These recurrent chromosomal abnormalities can be exploited as therapeutic targets. The deletion of chr8p in BC, for example, has been associated with perturbations in fatty acid and ceramide metabolism, favoring cell growth and invasiveness. Surprisingly, a drug response assay also showed that this specific aneuploidy confers a specific drug sensitivity profile. Recently, another study in liver cancer proved that the chr8p deletion sensitizes tumor cells to NUDT17-specific inhibitors [142]. It is expected that the increased knowledge of recurrent aneuploidies and their functional impact may guide the rational design of novel anticancer drugs and advance precision oncology.

### 4.3. Clinical Implications of CIN in Current Cancer Therapies

In addition to constituting potential therapeutic targets, several studies have shown that CIN and the resulting aneuploidy affect the response to current therapies, including radiotherapy, chemotherapy, and immunotherapy. The role of CIN and aneuploidy in sensitivity and resistance to these therapies seems to be context dependent. While moderate CIN might promote tumor heterogeneity and a favorable niche to develop resistance, higher rates of CIN have been related to treatment sensitivity [33,143,144]. For instance, a study showed that ionizing radiation led to mitotic chromosomal segregation errors and cell death [145]. Interestingly, reducing CIN and aneuploidy in in vivo models significantly increased the viability of irradiated cells, thereby inducing tumor radio-resistance. Another recent study also validated such findings, showing increased basal levels of CIN-sensitize cancer cells in patient-derived xenograft models [146]. Additional studies aiming to understand the role of specific proteins related to CIN in radiotherapy illustrate the importance of this phenomenon in cancer treatment [147,148].

Regarding chemotherapy, paclitaxel is one of the most studied examples of current drugs tightly linked to CIN [149]. Paclitaxel is a microtubule stabilizer agent belonging to the taxane class and is widely used in oncology for the treatment of breast, ovarian, and lung cancer, among others [149]. Although mitotic arrest was initially proposed as one of the principal mechanisms of action, multiple observations found that clinical concentrations of this agent were insufficient to produce mitotic arrest, but indeed led to abnormal mitotic spindles, chromosomal segregation errors, and cell death [150]. Furthermore, in a study conducted using a BC model, it was demonstrated that increasing abnormal divisions induced by paclitaxel through pharmacological or genetic modifications enhanced cytotoxicity [151]. Remarkably, both artificial methods of inducing CIN and pretreatment CIN levels modulated paclitaxel efficacy, suggesting a potential synergistic target and a predictive biomarker for drug response. Similarly, experiments assessing the radiosensitizer properties of docetaxel, another taxane agent, have shown that abnormal spindles and CIN are the main mechanisms of action of these agents [146]. Further studies in tumor chemoresistance have shown that CIN and aneuploidy may modulate the response to other commonly used antineoplastic agents, including cisplatin, anthracyclines, and vinca alkaloids [152,153,154].

Finally, the growing importance of immunotherapy in cancer has highlighted the central role of the tumor ecosystem, cancer heterogeneity, and CIN in cancer treatment. Two breakthroughs in this area are our best understanding of the molecular mechanisms underlying the immunosuppressive tumor microenvironment and the current capacity of tumor immunoediting in clinical settings [106,155]. While the complete panorama is still elusive, considerable progress has been made in understanding the relationship between CIN and immunity [156]. Amongst these actors, the cGAS-STING pathway, activated by CIN as described above, has been demonstrated to play a critical role in immunomodulation and possess long-term immunosuppressive tumor properties [157]. A recent study, for instance, found differential patterns of cGAS/STING expression and activation amongst BC subtypes [158]. These patterns were correlated with the number of PD-L1-positive tumor cells, suggesting diverse responses to immune checkpoint inhibitors. In line with these observations, preclinical data have shown that STING agonists improve the effect of immunotherapy and radiotherapy [159]. Another interesting subject of research in this topic is the impact of CIN in tumor evolution and immune escape. In this regard, for example, a study overexpressing Plk1 to increase CIN in a BC mouse model showed that tumor cells activated a senescence-associated secretory phenotype and upregulated PD-L1, thereby facilitating immune evasion [160]. Altogether, these results reflect the importance of CIN and tumor heterogeneity to understand and enhance cancer therapeutics.

## 5. Future Perspectives

Novel technologies and discoveries in cancer biology have illuminated our understanding of how CIN, aneuploidy, and CH impact tumor initiation, progression, and treatment. In addition to potentially being used in clinical settings, this knowledge has also opened new avenues of research. For instance, as mentioned above, one of the main challenges yet to be addressed in this field is how to quantitatively measure the CIN phenotype. Currently, despite many approaches being available, they vary widely in accuracy and feasibility [81,161]. In clinical oncology, where reliable and accurate biomarkers are needed, the development of methods to quantify CIN is of paramount importance. A successful example of such implementation in other settings is the homologous recombination deficiency (HRD) score for targeted therapy in BC and other tumors [162]. This score is based on multiple biomarkers, including oncogenic variants in homologous recombination repair (HRR) genes and genomic scars resulting from the deficiency in the HRR system. Similar to this approach, a robust CIN score might include several biomarkers, for example, expression signatures, genomic scars, and oncogenic variants in CIN driver genes. A potential ally in this endeavor is the implementation of machine learning (ML) and artificial intelligence (AI) methods. These methods have been successfully employed in cancer research and clinical oncology, and it is expected that the study of multivariable and complex processes such as CIN and CH can benefit from these techniques [163,164]. In this regard, two recent studies trained deep learning algorithms to predict HRD and the CIN phenotype from histologic slides with high accuracy [165,166]. Machine-learning-based techniques have also been used to identify potential CIN biomarkers and therapeutic targets aimed at CIN [167]. Furthermore, this group of techniques might be instrumental in integrating multiomics data, including expression, genomic, and clinical data, and studying regulatory networks controlling biological processes [168,169].

Methods able to dramatically change our study approach and understanding of the CIN phenotype and CH are single-cell sequencing-based techniques. In contrast to bulk multi-cell sequencing, these technologies rely on the isolation of cells from tissues and individual cell tagging, resulting in an unprecedented resolution level to explore complex cellular processes [170,171]. For example, scDNA-seq sequencing has been proposed as the most comprehensible method to assess the CIN phenotype in clinical samples [79]. While single-cell sequencing techniques are not currently used in clinical practice, a growing number of studies have used these techniques to investigate the intricacies and dynamics of the CIN phenotype and CH [97,143,172]. Two interesting studies to illustrate this approach were the identification of CIN as a driver of drug resistance and the use of scRNA-seq data to study cell–cell interactions in tumors [97,143]. In the first study, the researchers transiently induced CIN in cancer cell lines and exposed them to different therapeutic agents and conditions [143]. The authors then performed scDNA-seq and found that CIN-induced karyotypic heterogeneity and resistant populations developed recurrent chromosomal abnormalities. In the second study, the investigators performed single-cell transcriptomics in mice tumors derived from an orthotopically transplanted TNBC cell line with high and low CIN levels [97]. In addition to observing a positive correlation between CIN levels and an immunosuppressive tumor microenvironment, the authors developed and validated a tool to map cell–cell interactions, identifying the cGAS-STING pathway and the endoplasmic reticulum stress response as critical regulators of CIN-induced immune suppression. New developments in sequencing technologies, including spatially resolved transcriptomics, single-nucleus sequencing, and single-cell multiomics, will be useful to elucidate the mechanisms and effects of these events [173,174,175].

Finally, another important active area of research in this field is the development of in vitro and in vivo models to investigate CIN and related processes. Currently, CIN can be induced through both genetic and pharmacological methods in different models, hampering robust and comprehensive studies [79,151]. Furthermore, different chromosome engineering methods including Cre–lox-mediated recombination, transcription activator-like effector nuclease (TALEN), lentivirus, and CRISPR/Cas9 have been used to study recurring chromosomal abnormalities [45,107]. A recent study analyzed the recurrent TNBC loss of chr4p through a combination of lentivirus-mediated integration of chr4p genes and scRNA-seq in BC cell lines [45]. Intriguingly, the authors found that the overexpression of genes located in this locus, particularly C4orf19, suppressed proliferation in a context-dependent manner, supporting a genetic network rewiring process in cancer. Recently, new techniques based on transgenic kinesins and targeted assembly of ectopic kinetochores have been added to the repertoire of methods to induce chromosome-specific aneuploidies [176,177].

Another exciting field of research is the implementation of tissue engineering methods for the study of the CIN phenotype in the tumor microenvironment. For example, a recent study demonstrated that cells with high CIN levels formed larger tumors in nude mice and three-dimensional (3D) cultures [178]. Conversely, cells with low CIN grew faster in two-dimensional cultures. Surprisingly, the authors also found that karyotype heterogeneity was reduced in 3D cultures of cells with high CIN levels, suggesting an active process of selection for cells with growth advantages. Another interesting study that used patient-derived organoids from primary BC found that misaligned chromosomes are a major source of CIN in primary and metastatic BC [179]. These and other techniques aiming to investigate the causes and consequences of CIN will not only be critical for understanding tumor development and progression but also for translating basic research in cancer biology into clinical practice.

## 6. Conclusions

A growing body of evidence highlights the crucial role of the cellular mechanisms of CIN and clonal heterogeneity in tumor initiation and progression. Understanding these processes not only deepens our knowledge of tumor biology but also has significant clinical implications for BC and other malignancies. CIN, for instance, is a potential cancer biomarker, a susceptible target for novel therapeutic interventions, and a modifier of current cancer therapies. However, several challenges remain, particularly the lack of a standardized method for assessing CIN and CH. While various detection techniques exist, each has limitations. Advances in gene expression profiling, single-cell sequencing, and other emerging technologies have provided valuable insights into the origins and consequences of CIN and CH. Furthermore, recent studies using these approaches have provided new insights to explore the triggers and consequences of CIN and CH in BC. Moreover, exploring these features as therapeutic targets has the potential to improve cancer treatment strategies. Integrating novel methodologies in both research and clinical settings will be essential for refining detection approaches, enhancing therapeutic interventions, and ultimately improving patient outcomes.

## Figures and Tables

**Table 1:** Description of the advantages and limitations of different techniques for assessing the CIN phenotype.

Techniques	Advantages	Limitations
G-Banding Karyotype	Cell-by-cell evaluation of all chromosomes.	Requires dividing cells.
	Detection of complex rearrangements (operator dependent).	Does not detect small alterations (<5–10 Mbp).
	Low cost and availability.	Labor intensive.
Fluorescence In Situ Hybridization (FISH)	Cell-by-cell evaluation in interphase or metaphase nuclei.	Requires probes designed to target specific alterations.
	A large number of cells can be analyzed in a single assay.	Detection of individual alterations rather than whole-genome evaluation.
	Better resolution (~150 Kbp).	Labor intensive.
Spectral Karyotyping (SKY)	Cell-by-cell evaluation of all chromosomes.	Requires dividing cells.
		Requires specialized equipment.
	Better resolution for detecting complex rearrangements.	Labor intensive.
		High costs.
Array Comparative Genomic Hybridization (aCGH)	Genome-wide assessment of DNA gains and losses.	Assessment of a cell population rather than a single-cell resolution.
	Better resolution for detecting smaller alterations (>50–100 kb) (according to the resolution level).	Inability to detect chromosome alterations that do not result in copy number changes (e.g., balanced translocations).
	It does not require dividing cells.	Requires specialized equipment.
		Labor intensive.
Optical Genome Mapping (OGM)	Detection of balanced and unbalanced chromosome alterations, as well as submicroscopic alterations (>500 bp).	Assessment of a cell population rather than a single-cell resolution.
		Requires specialized equipment.
		Labor intensive.
		High costs.
Single-cell DNA Sequencing (scDNA-seq)	Cell-by-cell evaluation in interphase or metaphase nuclei.	It may require specialized equipment.
	A large number of cells can be analyzed in a single assay.	Complex data analysis.
	Genome-level resolution to detect chromosome alterations	High costs.
		Limited number of studies assessing its reliability.
Single-cell RNA Sequencing (scRNA-seq)	Cell-by-cell evaluation in interphase or metaphase nuclei.	It may require specialized equipment.
	A large number of cells can be analyzed in a single assay.	Detection of chromosome alterations is dependent on expression levels.
	High resolution to detect chromosome alterations.	Complex data analysis.
	Assessment of CIN gene expression signatures.	High costs.
		Limited number of studies assessing its reliability.
